# Application of Neurocomputing for Data Approximation and Classification in Wireless Sensor Networks

**DOI:** 10.3390/s90403056

**Published:** 2009-04-24

**Authors:** Amir Jabbari, Reiner Jedermann, Ramanan Muthuraman, Walter Lang

**Affiliations:** Department of Electrical Engineering, Institute of Micro sensors, Actuators and Systems (IMSAS), University of Bremen, NW1 Building, D-28359 Bremen, Germany

**Keywords:** Radial basis function, back propagation, wireless sensor network, distributed Data approximation and classification

## Abstract

A new application of neurocomputing for data approximation and classification is introduced to process data in a wireless sensor network. For this purpose, a simplified dynamic sliding backpropagation algorithm is implemented on a wireless sensor network for transportation applications. It is able to approximate temperature and humidity in sensor nodes. In addition, two architectures of “radial basis function” (RBF) classifiers are introduced with probabilistic features for data classification in sensor nodes. The applied approximation and classification algorithms could be used in similar applications for data processing in embedded systems.

## 1. Introduction

There are different means of transporting products between cities and countries worldwide. According to the type and importance of the transported products, certain requirements are considered in the selection and supervision of transportation systems [1]. The use of wireless sensor networks to record environmental conditions such as temperature and humidity during the transport of sensitive goods and products has increased considerably [2,3]. After measuring environmental conditions, data are sent for processing and decision-making; in advanced transportation systems, key decisions are made in measurement systems in a distributed manner [4]. The use of distributed data processing techniques increases the autonomy and reliability of transportation systems. This allows further decisions to be made based on the current condition of the goods. The “intelligent container” is an example of an intelligent transportation system that features distributed data processing [5]. In recent years, several data processing and analysis techniques have been developed. Typically, the processing algorithm consists of both an approximation mechanism and a classification algorithm. The approximation theory concerns the approximation of unknown functions or parameters according to known functions or parameters [6].

Different approaches exist to approximate data, including stochastic approximation, polynomial interpolation, differential and integral equations, “least squares”, and “neural network” [7]. Furthermore, for data classification and inference, different algorithms such as fuzzy, neural network and the hierarchical approach can be applied [8–10]. The artificial neural network (ANN) is a knowledge-based approach with several applications in engineering, economics, and transportation industries [11]. There are two main ANN approaches for parameter approximation, including the “radial basis function” (RBF) approach and the “multi-layer perceptron” (MLP) approach that incorporates the “backpropagation” technique [12]. RBF is a local approximator that yields greater accuracy in local purposes, while MLP is a more appropriate choice for global approximation [13]. Backpropagation leads to either a linear or nonlinear mapping between the input and output by an algebraic activation function. Backpropagation requires a certain number of input sets to train the network to initiate the approximation. The number of input sets, the accuracy of the training, and the parameters of the network greatly influence the accuracy of the approximation. The application of the traditional backpropagation technique to embedded systems could generate problems due to the constraints of memory size, processing capability and energy required for the calculation. To overcome these limitations, in this study the entire network is continuously updated for training and data approximation solely by using a limited number of neurons and samples.

Data classification is a secondary neural network application that is especially useful when the data classes are only partially known [14,15]. Moreover, due to the employment of probabilistic features, making decisions regarding class borders is possible. The development of probabilistic neural networks is based on training the network according to data classes; the new data is classified according to the recently obtained “probability density function” (PDF) [16].

**Figure 1.**
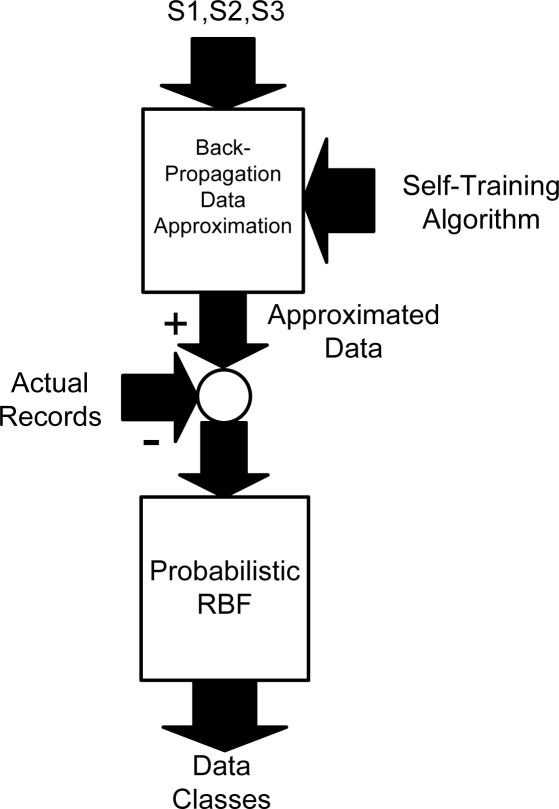
ANN for data approximation and classification.

In our study, to wirelessly process the data, the data are first approximated by a dynamic backpropagation mechanism and then classified by a probabilistic radial basis function (RBF) network implemented on a wireless sensor network, seen in Figure 1. Using two different ANN architectures leads to flexibility and higher accuracy of approximation and classification mechanisms. The data approximation is carried out for temperature and humidity records of different positions in a food transportation truck. After the data are approximated, they are compared with current values, thereby generating so-called approximation residuals. Finally, according to the structure of the probabilistic RBF classifier, the data are classified into one of several predefined classes. The defined classes are used to evaluate reliability of the records in wireless sensor network. Therefore, the applied backpropagation algorithm approximates the records of each node which is processed by an RBF classification network to detect any abnormality in wireless sensor network.

## 2. Related Works

Presently, knowledge-based approaches are applied to intelligent transportation. ANN-based diagnosis, real-time traffic signal control, and road signal analysis are some applications of ANN found in transportation systems [17,18]. An automated food inspection system is a further application for use in intelligent food transportation industries [19]. There are different approaches for processing data in wireless sensor network. Collaborative information processing in wireless sensor network is one of the examples [20]. The processed data could be used for routing or any local decision making in wireless sensor network [21]. Also, data processing increases the performance of the network [22]. The selected processing technique should be accurate and energy efficient [23]. When wireless sensor networks are utilized, neural networks could also be implemented for data fusion [24,25]. Various studies have examined the use of data fusion techniques in measurement systems to process data in order to evaluate the reliability of sensor records [26].

The data must first be approximated. Different techniques are applied in wireless sensor network for data approximation depending on application. Then, the approximated data could be used either for data fusion or fault diagnosis purposes [27]. The approximation technique is established on either linear or nonlinear mapping between sensor records [28]; the applied technique could give a prediction about records of any sensor node in wireless sensor network. Backpropagation could be an appropriate choice, because this method establishes a nonlinear mapping between data, preferable to linear approaches [29]. After a training period using a limited amount of data from all sensor nodes, the network is ready to approximate new data from each sensor node according to new data of the others. However, the neural network requires training samples and a certain evolution time to sufficiently map data to achieve an accurate approximation. For continuous data training and approximation, the training set and network architecture could be dynamically changed and updated [30].

Probabilistic features also make the neural network an important model for data classification [31]. Depending on the application, different neural network variations are employed for use in data classification [32]. The probabilistic neural network (PNN) is a well-known approach comprised of both a competitive neural layer and a hidden layer, which includes various radial basis functions [33]. In order to classify data, the competitive layer selects the highest value among the outputs of the hidden layer in the network.

## 3. Theoretical Concepts

**Figure 2.**
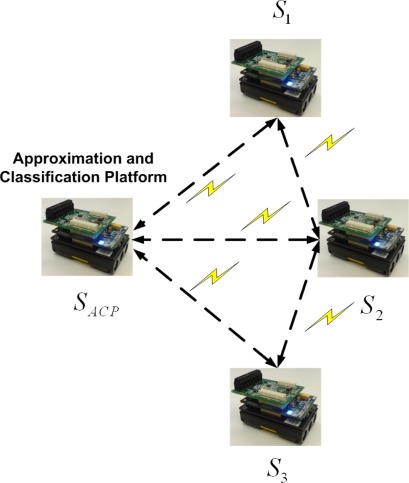
Architecture of the wireless sensor nodes.

As mentioned, we propose an application of two joint mechanisms for wireless data approximation and classification in food transportation. For this purpose, the Imote2 kit is used to record temperature and relative humidity and to process the recorded data. The kit consists of three main components, including a radio/processor board, a sensor board, and a battery board [34,35]. All algorithms are executed in a wireless sensor network. One of the sensor nodes is selected as an “approximation and classification platform” (ACP) which works as “approximation platform” (AP) and also “classification platform” (CP). It is possible to assign the task of data approximation and classification to each individual sensor node, but the resultant increased data transfer and inference would raise energy consumption. Each temperature and humidity record is sent and received via the Imote2’s CC2420 radio, and the data obtained from all nodes are processed on the ACP. Figure 2 illustrates the architecture and location of each sensor node. There are three sensor nodes that communicate with the ACP.

The sensor nodes are positioned in different locations throughout the inside of the food transportation truck. A reefer unit inside the truck establishes the desired environmental conditions [36]. According to the set points, the reefer unit is able to adjust the temperature by on/off cycles. Sensor “*S*_1_” records the temperature and humidity of the reefer unit. Sensors “*S_ACP_*” and “*S*_2_” are located in the middle of the truck, and “*S*_3_”is in front of the door, farthest from the reefer unit. The task of finding the best sensor positions for optimum data approximation will be addressed in the next section of this paper.

The sensor nodes are programmed in Microsoft^®^ .NET Micro Framework using Visual Studio. Compared to the .NET Framework, the .NET Micro Framework is limited [37], requiring that all neural network functions be defined in the programming environment. Thus, implementing a simpler, optimized approximation algorithm is vital due to processing time and energy constraints. Additionally, the data approximation and classification mechanism should be accurate enough to evaluate data directly in the sensor network. Also, considering the limitations in providing the well-determined training set, the standard backpropagation algorithm is not applicable for data approximation; to train the network just a few records of each sensor nodes could be used (instead of using large number of data) [38]. For this purpose, the traditional static backpropagation algorithm could be simplified and dynamic. After training, the network initiates approximation. To approximate the new values, the network continues to train by sliding over the last few samples. The applied self-training refers to the ability of the ACP to autonomously train to approximate data in real conditions. Instead of feeding a large number of samples to the network, only four samples from each sensor node are utilized to train the network. In other words, only the first four records of each sensor node are used as input vectors to train the approximation mechanism. During the training phase, the network maps the input vectors to the first four records of each sensor node currently being approximated (named the “under approximation sensor node”). Thereafter, the 5th record of each “under approximation sensor node” is approximated according to the 5th sample of the other sensor nodes as the input to the network for temperature and humidity data. Then, after approximating the fifth sample, the reliability of record is checked using the classification mechanism. In other word, if the difference between actual and approximated value shows significant deviation, the mechanism could investigate the reason; if the network could detect any fault, then the 3D approximation stops (3D denotes approximation of data for each sensor node using the other three nodes) and the records of the faulty sensor doesn’t contribute in next data approximations; then 2D (2D denotes approximation of data for each sensor node using two other faultless nodes) approximation continues prediction which could also switch even to 1D approximation.

Otherwise, when the actual record of 5th sample is reliable, the last four samples (namely, the 2nd, 3rd, 4th, and 5th samples) are used for training and to approximate the 6th value using the 3D approximation algorithm and it continues to predict next values. This procedure, called the sliding backpropagation algorithm, depends solely on the last four reliable values. By gradually receiving each piece of data from the other sensor nodes, the approximation mechanism updates all of the network weights; this sliding “weight update” stems from the “least squares” (LS) approximation approach that uses members of a small group of related previous equation sets [39]. The benefits of using the sliding backpropagation algorithm will be discussed along with its results.

### 3.1. Approximation mechanism

**Figure 3.**
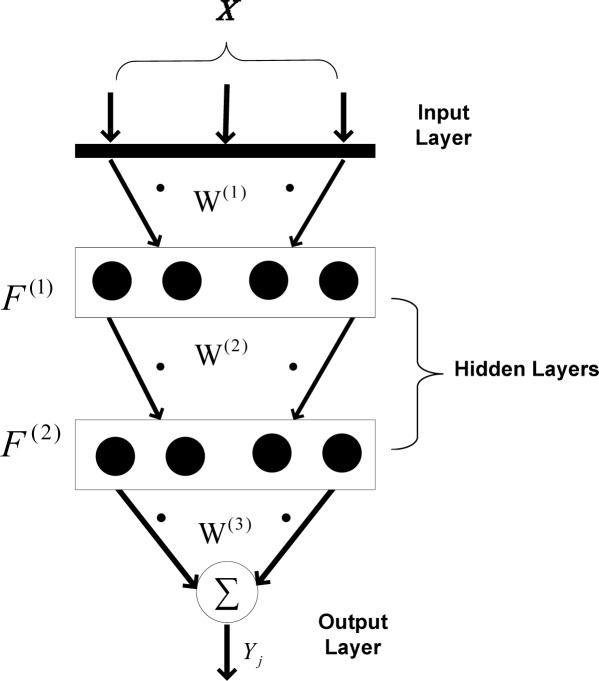
Applied ANN approximation mechanism.

Figure 3 shows the backpropagation mechanism used to approximate data in the applied wireless sensor network. As shown, two hidden layers are taken into account, while the output layer merely sums the weighted data. There are four neurons in each hidden layer. Using two hidden layers increases the nonlinear mapping feature between input pattern and target. Also, it has been tested that using four neurons in each layer has optimum performance for data approximation, when only four sensor nodes are used for data approximation. Using fewer neurons decreases the mapping performance and using more neurons increases the calculation time considering the fact that the results are not improved compared to current architecture.

In (1), $H_{h_{1}}^{\left(1\right)}$ is the weighted input of the i-th neuron in first hidden layer, $w_{{\mathrm{ih}}_{1}}^{\left(1\right)}$ is the weight vector between the i-th input and the *h*_1_-th neuron in the first hidden layer, and *x_i_* is the i-th input layer element. After $F_{h_{1}}^{\left(1\right)}$ is calculated, the output of each neuron is obtained using (2).

(1) $$\begin{array}{l} H_{h_{1}}^{\left(1\right)}=\sum_{i=1}^{3}w_{{\mathrm{ih}}_{1}}^{\left(1\right)}x_{i}\\ h_{1}\;=\;1,...,4 \end{array}$$

(2) $$\begin{array}{l} F_{h_{1}}^{\left(1\right)}(H_{h_{1}}^{\left(1\right)})={\left(1+e^{\left(-H_{h_{1}}^{\left(1\right)}\right)}\right)}^{-1}\\ h_{1}=1,...,4 \end{array}$$

$F_{h_{1}}^{\left(1\right)}$ is the output of *h*_1_-th neuron in the first hidden layer after the outputs of all hidden neurons in first layer are calculated. In a similar manner, the weighted inputs ( $H_{h_{2}}^{\left(2\right)}$) and neuron outputs ( $F_{h_{2}}^{\left(2\right)}$) of the second hidden layer are calculated. In equations (3) and (4), $w_{{\mathrm{ih}}_{2}}^{\left(2\right)}$ denotes the weights between the first and second hidden layers.

(3) $$H_{h_{2}}^{\left(2\right)}=\sum_{i=1}^{4}w_{{\mathrm{ih}}_{2}}^{\left(2\right)}F_{i}^{\left(1\right)}$$

(4) $$\begin{array}{l} F_{h_{2}}^{\left(2\right)}(H_{h2}^{\left(2\right)})={\left(1+e^{\left(-H_{h_{21}}^{\left(2\right)}\right)}\right)}^{-1}\\ h_{2}=1,...,4 \end{array}$$

By calculating and summing the outputs of each neuron in the second hidden layer, the output of network (*Y_j_*) is calculated (5). The output vector refers to the temperature and humidity of each sensor node that is being approximated at that time (the so-called “under approximation sensor node”).

(5) $$Y_{j}=\sum_{i=1}^{4}w_{i}^{\left(3\right)}F_{i}^{\left(2\right)}$$

To train the network, a “gradient descent” algorithm is applied to minimize the error function (E) between the desired and actual outputs of the network.

(6)

In (6), D_j_ and Y_j_ refer to the desired and actual outputs. The output error e_j_(k) is calculated for each sample (k) and is defined as the difference between the desired and network outputs in the output layer. The output error is minimized by updating the weight values towards decreasing the error function. The weight changes are proportional to the negative gradients of the error function and current weights (according to equation (7), where η is the learning rate):

(7) $${\Delta W}^{\left(3\right)}=\;-{\eta \nabla W}^{\left(3\right)}E$$

After calculating the weight changes, the weights between the second hidden layer and the output layer are updated. Similarly, the remaining weights are consequently updated according to (8):

(8) $$\begin{array}{l} W^{\left(3\right)}(k+1)=\;W^{\left(3\right)}(k)+{\Delta W}^{\left(3\right)}\\ W^{\left(2\right)}(k+1)=\;W^{\left(2\right)}(k)+{\Delta W}^{\left(2\right)}\\ W^{\left(1\right)}(k+1)=\;W^{\left(1\right)}(k)+{\Delta W}^{\left(1\right)} \end{array}$$

Like the applied ANN algorithm, the LS approach relates the records of the “under approximation sensor node” and the last four records of other sensor nodes by an over-determined matrix as shown in (9):

(9) $$\begin{array}{l} \left[\begin{array}{l} S_{3}(t)\\ S_{3}(t+1)\\ S_{3}(t+2)\\ S_{3}(t+3) \end{array}\right]=\\ \left[\begin{array}{l} \begin{array}{ccc} S_{\mathrm{ACP}}(t) & S_{1}(t) & S_{2}(t)\\ S_{\mathrm{ACP}}(t+1) & S_{1}(t+1) & S_{2}(t+1)\\ S_{\mathrm{ACP}}(t+2) & S_{1}(t+2) & S_{2}(t+2) \end{array}\\ \begin{array}{ccc} S_{\mathrm{ACP}}(t+3) & S_{1}(t+3) & S_{2}(t+3) \end{array} \end{array}\right]\cdot K \end{array}$$

In (9), the current and previous values of parameters *S_ACP_*, *S*_1_ and *S*_2_ are recorded and used to find the approximation coefficients ( $K={\left[\begin{array}{ccc} k_{\mathrm{ACP}} & k_{1} & k_{2} \end{array}\right]}^{T}$) that are calculated by solving the over-determined set [40]. With the new values of approximation parameters (*S_ACP_*, *S*_1_ and *S*_2_) and the updated approximation coefficients (*K* vector), a new value of *S*_3_ (the value of the “under approximation sensor node”) is calculated.

(10) $$\begin{array}{l} S_{3}(t+4)=\\ \left[\begin{array}{ccc} S_{3}(t+4) & S_{3}(t+4) & S_{3}(t+4) \end{array}\right]\cdot K \end{array}$$

This procedure continues and the coefficients are constantly updated based on the last four instantaneous equations. To calculate the values of the other sensors, the same algorithm is applied by replacing the sensor names and values.

### 3.2. Classification mechanism

The simplified probabilistic RBF algorithm is used to classify data. The probabilistic RBF algorithm is applied primarily to calculate the “probability density function” for assigning the network output to predefined classes. Sometimes, according to reading errors or weaknesses in approximation, the approximation exceeds the desired accuracy in wireless sensor network. Further research is needed to determine if this excess stems from an inaccurate approximation, or from a fault or failure inside the system that causes error and uncertainty. For data classification, an alternative could be using the classical RBF classifier including three output layers, however, for increasing the accuracy of data classification, more training data should be used in this case. In classical RBF, when the number of training sets is not enough, the algorithm could infer incorrect classes.

**Figure 4.**
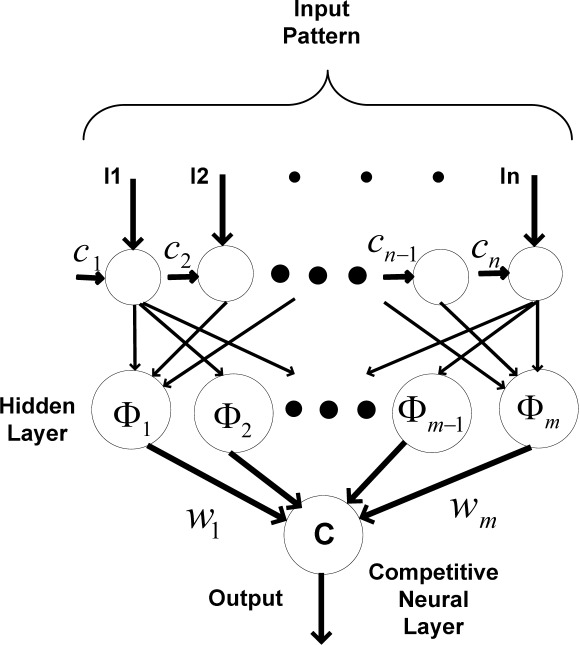
Applied RBF classification mechanism.

Probabilistic neural networks are feed-forward networks derived from “Bayesian decision networks” [41]. Probabilistic neural networks estimate the “probability density function” for each class based on the given training samples. Figure 4 shows the implemented probabilistic RBF network, including a nonlinear transfer function of neurons located in each hidden neuron.

The training algorithm of the RBF network is a “Gradient descent” algorithm which is applied for mapping input set to predefined classes. An appropriate number for center should be assigned to each neuron in the hidden layers. There are different choices for assigning number of centers. The number of centers could be equal, less or more, than number of inputs.

When the number of input sets is more than the number of centers, it takes more time for the calculation, because the calculated weight values needs training algorithms for updating all weight values each time. The assumption of considering fewer input sets than centers is quite unrealistic. In this research, equal numbers of centers and input sets are considered. Thus, the “Gradient descent” training algorithm is not necessary to update the weight values each time; it is applied whenever the training error exceeds the training accuracy. The training set must be a thorough representation of the data. Probabilistic neural networks handle data that have spikes and points outside the norm better than the classical RBF network.

To train the probabilistic neural network, the radial basis function Φ*_i_* is calculated according to the assigned center. Also, preliminary weights between the hidden layer and the output layer are calculated. After calculating the weighted input pattern, a nonlinear function is applied in the hidden layer. Thereafter, the weighted outputs of each hidden neuron in the hidden layer are calculated (*O_i_*);

(11) $$\begin{array}{l} O_{i}=w_{i}\Phi_{i}(\left\|l_{i}-c_{i}\right\|)\\ i=1,2,3,...,m \end{array}$$

In (11), *l_i_* is the i-th element of the input vector of the probabilistic RBF network, *c_i_* is the i-th assigned center, ‖ · ‖ is the Euclidean norm, Φ*_i_* is the i-th nonlinear function, *m* is the number of neurons in the hidden layer, and *w_i_* is the i-th weight applied to the output of the i-th hidden neuron.

Different nonlinear functions, such as “multi-quadratic” and “spline”, are used to calculate the output of each hidden neuron. In this paper, the “Gaussian function” is used as the nonlinear function for the hidden neurons, which results in a nonlinear mapping between the inputs and classes.

The output layer compares the data using the competitive neural layer which leads to selecting the highest value.

(12) $$\begin{array}{l} O_{k}(l)\geq O_{i}(l)\\ i=1,2,3,.....,m \end{array}$$

In (12), *O_k_* refers to the highest value greater than all other output values from hidden neurons. The “probabilistic neural network” is immediately trained, although the execution time is slower. The training set must be a thorough representation of the data. This network handles data, even those with spikes and points outside the norm, more accurately than other neural networks.

## 4. Experimental results

### 4.1. Data approximation

**Figure 5.**
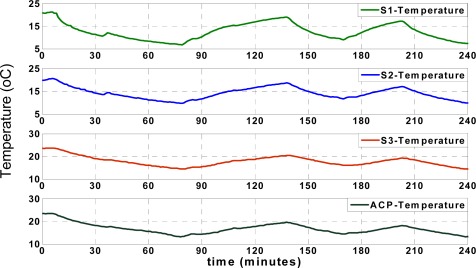
Actual temperature of reefer unit and sensor nodes.

Figure 5 shows the actual (recorded) temperatures of four different locations, including *S_ACP_*, *S*_1_, *S*_2_ and *S*_3_, as shown in Figure 2. The temperature records of “*S*_1_” show the temperature of the reefer unit, which fluctuates over time according to the unit’s on/off cycles. The reefer unit could cool down or warm up the truck according to set points, illustrated in Figure 5, which shows the arbitrary set points used to test the performance of the applied approximation mechanism between 5 and 25 °C. All temperature and humidity records in different positions are affected by the reefer temperature.

**Figure 6.**
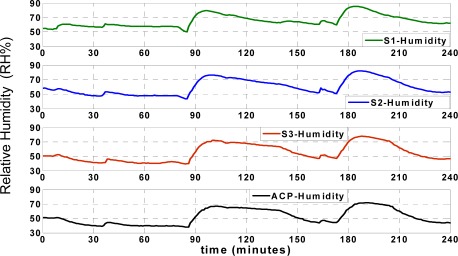
Actual humidity of reefer unit and sensor nodes.

In this research, first the “data request” message from ACP is sent for each sensor node every 2 seconds respectively according to predefined scenario; every two seconds the ACP collects data from each node and it takes 6 seconds to collect data from all sensor nodes by ACP. It means that the ACP starts 3D approximation and classification after collecting data from all three sensor nodes (3D denotes approximation of data for each sensor node using the other three nodes). Otherwise, the algorithm is able to switch to 2D (2D denotes approximation of data for each sensor node using two other faultless nodes) or even 1D approximation depending on the available received records in ACP [36]. Also, data collision is prevented in this application due to the applied “data send/receive” scenario. The humidity records of the aforementioned points (*S_ACP_*, *S*_1_, *S*_2_ and *S*_3_) are illustrated in Figure 6;

The ACP receives the first four temperature and humidity records of different points as input vector for training. The network creates a mapping between the input and target vectors during the training phase. The target vector corresponds to the previous and current values of the “under approximation sensor node”. Three possible ways to approximate data include:

- Mapping (*T*_1_, *T*_2_, *T_ACP_*, *H*_1_, *H*_2_, *H_ACP_*) to (*T*_3_, *H*_3_) to approximate the temperature and humidity of “node 3” by ACP.
- Mapping (*T*_1_, *T*_3_, *T_ACP_*, *H*_1_, *H*_3_, *H_ACP_*) to (*T*_2_, *H*_2_) to approximate the temperature and humidity of “node 2”.
- Mapping (*T*_2_, *T*_3_, *T_ACP_*, *H*_2_, *H*_3_, *H_ACP_*) to (*T*_1_, *H*_1_) to predict the temperature and humidity of “node 1”.

The residual term is defined as the difference between the actual data and the approximation. This residual could be calculated for both temperature and humidity records individually:

(13) $$\left\{ \begin{array}{l} \Delta T=T_{\mathrm{Actual}}-T_{\mathrm{Network}}\\ \Delta H=H_{\mathrm{Actual}}-H_{\mathrm{Network}} \end{array}\right.$$

**Figure 7.**
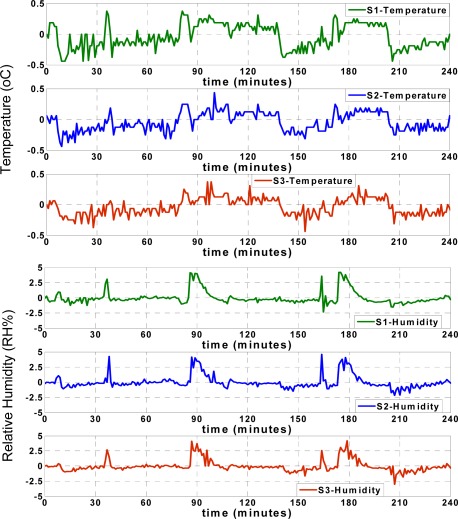
Approximation residual of each sensor node.

*T_Actual_* and *H_Actual_* denote the instantaneous actual recorded temperature and humidity. *T_Network_* and *H_Network_* are the approximated temperature and humidity values of each sensor node. The temperature and humidity residuals (*ΔT*, *ΔH*) are shown in Figure 7.

The accuracy of approximation is determined according to the experimental results in normal situation when the records are reliable. The approximation residual doesn’t exceed sum of the maximum reading error and training error. For example, the maximum reading error of temperature sensor is ±0.3 °C and the maximum training error is less than ±0.15 °C (sum is ±0.45 °C). The maximum ANN approximation error is considered ±0.5 °C which is observed in experimental results.

The maximum reading error of humidity sensor is ±2.5 RH% and the maximum training error is less than ±2RH% (sum is ±4.5 RH%). The maximum ANN approximation error is considered ±5 RH%. As seen in Figure 7, depending on the instantaneous approximation values, the approximation residual varies between ±0.5 °C for temperature records and ± 5 RH% for humidity records.

The accuracies of the approximations of the temperature and humidity records are compared with accuracies of the approximations using the pure LS approach. For a more precise comparison, the “root mean squared errors” (RMSE) of the temperature and humidity approximations are calculated for each sensor node using the same actual values that were used in the sliding backpropagation approximation [42].

(14) $$\mathrm{RMSE}(Y_{\mathrm{App}.})=\sqrt{\frac{\sum_{i=1}^{n}{\left(Y_{\mathrm{Actual}}-Y_{\mathrm{App}.}\right)}^{2}}{n}}$$

*Y_Actual_* and *Y_App._* are the actual and approximated values that could be assigned to either the instantaneous temperature or humidity values; *n* denotes the number of approximated records.

**Figure 8.**
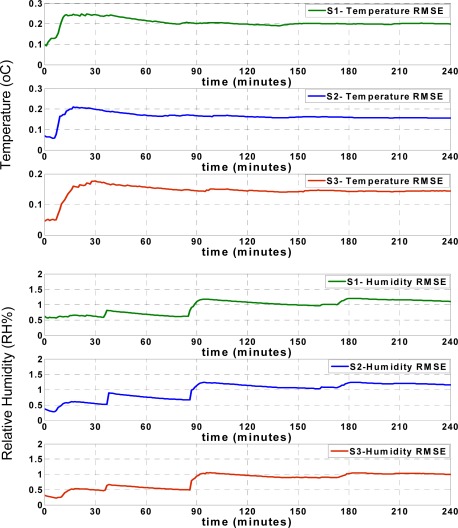
RMSE of ANN approximations.

Figure 8 shows that the RMSE of the temperature records for each sensor node increases during the first minutes of approximation, and afterwards remains mostly steady. The humidity RMSE values indicate an especially prominent increase when the approximation values show significant deviation from the actual values, as seen in Figure 7. However, similar to the temperature RMSE, the humidity RMSE changes slightly and after some time, remains mostly steady. These figures show that both the RMSE and approximation accuracy of the sensor nodes differ depending on the positions of sensor nodes in the truck, which will be subsequently discussed.

**Figure 9.**
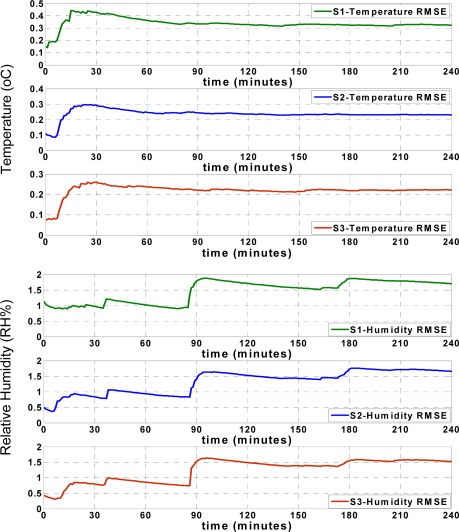
RMSE of LS approximations.

Figure 9 shows the approximation accuracy of the LS approach. By comparing the RMSE values found in Figures 8 and 9, the apparent accuracy of the new sliding backpropagation is higher than the accuracy found using LS. As previously mentioned, the approximation accuracy of the temperature records is within ±0.5 °C using the ANN approach, while the approximation accuracy is within ±1 °C using LS. For relative humidity, the approximation accuracy is within ± 5 RH% using ANN and within ± 10 RH% using LS. The results show that the newly introduced ANN approach performed better than the pure LS approach.

### 4.2. Sensor node allocation

**Figure 10.**
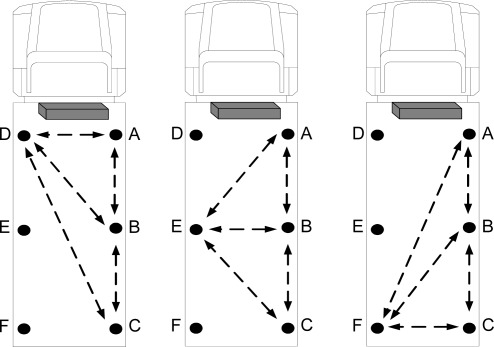
Allocation of approximation platform.

As previously mentioned, any temperature or humidity change found in the various positions of the truck is related to the reefer unit. A primary research task is to find the optimal position for the data approximation platform (AP). Three main alternatives are available as shown in Figure 10.

There are 6 zones, labeled A through F, which could be used to place sensor nodes. Previously, the AP was placed in the middle of the truck for data approximation. Currently, discovering the location exhibiting optimum performance is investigated.

For this purpose, all three alternatives were tested by positioning the three sensor nodes as three individual approximation platforms. Thus, the following three main cases were tested:

1. D as approximation platform for A, B, and C;
2. E as approximation platform for A, B, and C;
3. F as approximation platform for A, B, and C.

Then, the approximation residual and RMSE of each sensor node was calculated for each individual case. Finally, the average approximation residual was calculated separately for each of the three cases. This average returns the arithmetic mean of the arguments as the “average root mean squared error” (ARMSE). According to (14), the *RMSE*(*S_i_*) refers to the “root mean squared error” of the data approximation for each sensor node by the applied approximation platform (15):

(15) $$\mathrm{ARMSE}(S)=\frac{\sum_{i=1}^{3}\mathrm{RMSE}(S_{i})}{3}$$

**Figure 11.**
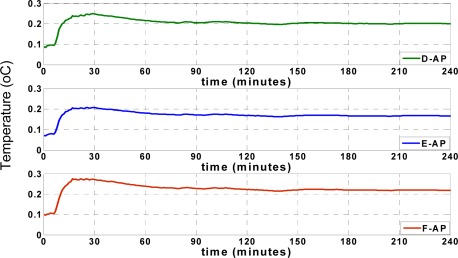
Performance of different approximation platforms (Temperature).

Figure 11 shows the ARMSE of each of the three tested cases for temperature records when the approximation platform (AP) was located at points D, E, or F.

**Figure 12.**
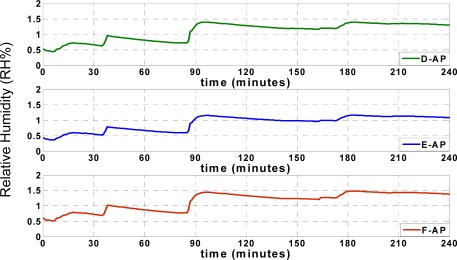
Performance of different approximation platforms (Humidity).

Figure 12 illustrates the ARMSE of the humidity approximation records when the AP was tested at points D, E, and F. The results prove that positioning the approximation platform at position E in the middle of the truck results in optimal performance compared to points D and F. Also, positioning the approximation platform at point F exhibits inferior performance compared to the other points. Optimal performance at point E is due to the result of increased correlation between the sensor nodes in the middle of the truck and the other nodes at different locations inside the truck. When D is selected as AP, a high correlation between data from points A and D leads to a very accurate approximation of temperature and humidity at point A. In addition, the data approximation at point B is sufficiently accurate, although the approximation of data at point C is not as accurate as at points A and B. The opposite occurs by selecting point F as AP, which improves the approximation accuracy of point C. However, the approximation accuracy at point A is not as great as at points B and C.

Finally, by using point E as AP, the approximation accuracy at point B increases and the accuracies at points A and C suffice. Thus, the optimal position for data approximation is in the middle of the truck at point E. The temperature and humidity changes at the farthest points, such as point C, are not instantaneously synchronized with the reefer unit, so that the accuracy of data approximation when the AP is located at point D is greater than when the AP is located at point F.

### 4.3. RBF Classification mechanism

After approximating temperature and humidity, an evaluation of the reliability of the records is necessary. The approximation residual and RMSE were used to detect any abnormal data change due to faults or failures in the wireless sensor network, such as battery problems or accidental door openings in truck. However, the approximation residual seldom exceeds the approximation accuracy in the absence of any fault or failure due to inaccurate sensor readings or insufficient data mapping. When the approximation residual exceeds the approximation ranges, the cause of this excess, such as a fault or failure in the system or weakness in approximation, must be determined. Three main residual ranges are defined to evaluate the reliability of the records. If the absolute value of the residual is used, the following cases could be defined for temperature and humidity approximation residuals:

1. Normal situation: 0 ≤ |Δ*T*| ≤ 0.5 (°C), 0 ≤ |Δ*H*| ≤ 5 (RH %);
2. Unknown situation: 0.5 ≤ |Δ*T*| ≤ 1 (°C), 5 ≤ |Δ*H*| ≤ 10 (RH %);
3. Abnormal situation: 1 ≤ |Δ*T*| (°C), 10 ≤ |Δ*H*| (RH %).

These cases are not especially accurate at the borders of classes, in which case the use of probabilistic features is required. Using probabilistic features could clarify the temperature and humidity residuals at the borders of the classes. The probabilistic RBF network is trained by samples which have the statistical requirements of the aforementioned cases, including:

1. Class 1: Normal;
2. Class 2: Unknown;
3. Class 3: Abnormal.

**Figure 13.**
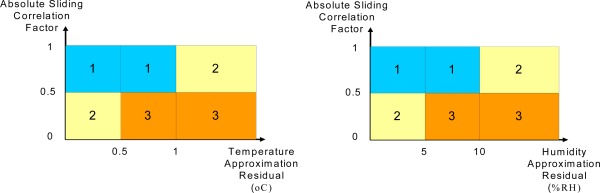
Classification range.

After training, the probabilistic RBF network is mapped to one of the classes instead of any new given input. Figure 13 shows the area of the classes according to the relationship between the residuals and the correlation factor between the recorded data and the ACP. The correlation factor is computed by (16);

(16) $$\mathrm{Corr}(X_{1},X_{2})=\frac{\sum (x_{1}-{\bar{x}}_{1}).(x_{2}-{\bar{x}}_{2})}{\sqrt{\sum {\left(x_{1}-{\bar{x}}_{1}\right)}^{2}.\sum {\left(x_{2}-{\bar{x}}_{2}\right)}^{2}}}$$

In (16), *Corr*(*X*_1_, *X*_2_) returns the correlation factor between sets *X*_1_ and *X*_2_ including their elements (*x*_1_ and *x*_2_) and average values (*x̄*_1_and *x̄*_2_) [43]. To increase the sensitivity to data change, according to the structure of the sliding backpropagation technique, the correlation factor uses the last four values each time. By receiving new data, the correlation function slides over samples. Thus, the sliding correlation function is highly dependant on the last four values. The absolute value of the sliding correlation factor is used in this paper.

As shown, temperature and humidity are evaluated separately and the results are combined. Although the temperature and humidity are not independent variables but the temperature and humidity sensors could be defective separately on sensor board and the main task of the approximation and classification algorithm is detecting any abnormal condition in wireless sensor network. Figure 13 shows that when the absolute sliding correlation factor is high, the normal and unknown situations are classified as 1st class, but when the function exhibits smaller values, the unknown area and abnormal situations are classified as 3rd class. The other cases are categorized as class 2, which means the mechanism requires more samples for decision-making.

**Figure 14.**
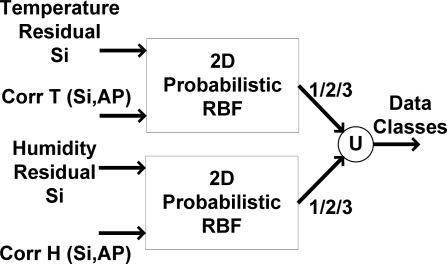
2D RBF classification diagram.

The applied 2D RBF mechanism is seen in Figure 14. According to Figure 14, by using the absolute sliding correlation factor of recorded data with AP and the approximation residual (as 2 dimensions of input vector), the class of recorded data is obtained using probabilistic features. The function (identified by U) denotes combining the results of the applied classification mechanism for temperature and humidity sensors, respectively.

Two different 2D RBF architectures are used because it is assumed that sensor defection could occur separately for either the temperature or humidity sensors. The results of the classification of the temperature and humidity data are combined at the end of the classification process. The networks are trained using Tables 1 and 2 for temperature and humidity classification, respectively, in the ACP. The training data are provided from real records that have the statistical requirements of defined classes. As shown, instead of mentioning border data, such as 0.5 as the correlation factor, only the accurate data that has the statistical features of each class is used to train the network. The network could classify any new data by employing probabilistic features. In this case, one of the three mentioned classes is assigned for any temperature as well as humidity record.

A further alternative to classify data employs a 3D RBF network only in unknown areas in which the absolute approximation residual exceeds the limits but remains less than 1 °C for temperature and 10% RH for humidity records (0.5 ≤ |Δ*T*| ≤ 1 (°C) and 5 ≤ |Δ*H*| ≤ 10 (RH%)). So, the applied 2D classifier is the main classifier and the 3D mechanism is only used for clarifying the actual situation of records in unknown area. This algorithm is established based on the relationship of correlation factors between sensors A, B, and C in the truck. In other words, the residual is calculated, and if it lies in an unknown area, the correlation factors between the sensors are considered.

**Figure 15.**
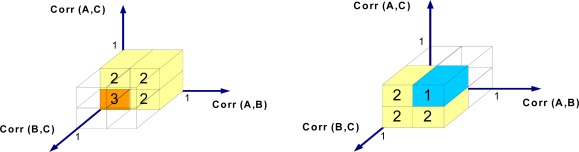
Classification range.

Figure 15 shows the 3D relationship between the absolute sliding correlation factors. According to Figure 15, when the absolute sliding correlation factors between all sensor nodes are high, the situation is classified as class 1 (normal). In the abnormal case in which all absolute sliding correlation factors are small, the records are unreliable. All other cases are classified as class 2, which demands more samples for decision-making.

**Figure 16.**
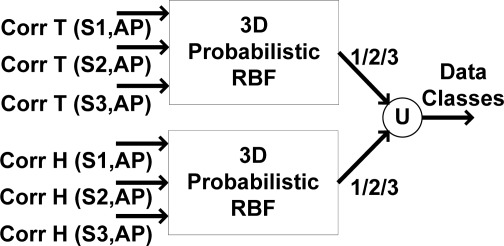
3D RBF classification diagram.

Figure 16 shows the applied 3D RBF network, which functions based on the absolute sliding correlation factor between sensor nodes. When the approximation residual exceeds the approximation accuracy, the area remains unknown. By using this RBF classifier, the classes of sensor nodes are clarified.

The training data in Table 3 are used to train the 3D RBF classifier. Like Tables 1 and 2, only the accurate data for each data class is used to train the network, and the data will classify any new given data into one of the classes. As previously mentioned, the 2D probabilistic RBF classifiers could classify all data, although the 3D probabilistic classifier is used solely to classify data in the unknown region. As mentioned, according to a sufficient correlation of the sensor nodes in the middle of truck with the other nodes in a normal situation, this could be used as the classification platform. The optimal placement of the approximation and classification platform (ACP) lies in the middle of the truck, where the applied approximation and classification algorithms are used together to evaluate the reliability of the temperature and humidity records.

## 5. Conclusions

In this paper, a neurocomputing approach was introduced and implemented for data approximation and classification. First, an optimized backpropagation network was defined for temperature and humidity approximation in a wireless sensor network. The results proved that the new applied approximation mechanism could predict the data more accurately than the “least squares” (LS) method. In addition, the goal of finding the optimal position of the approximation platform was discussed. The best position for this platform is in the middle of the truck, where the average “root mean squared error” (ARMSE) of data approximation is minimized. This position also correlates sufficiently with the other nodes to be used as a classification platform as well. To evaluate the reliability of the records, two types of probabilistic radial basis function networks were introduced. The networks were trained with accurate input sets and different classes. By feeding new input, the probabilistic classifiers were able to classify the data. The applied mechanism is applicable in similar projects for the purpose of data fusion in wireless sensor networks.

## Figures and Tables

**Table 1.** Training 2D RBF classifier for temperature records.

**Case** [Approximation Residual (°C), Absolute Correlation with AP ]	**Class**
(0.25,0.25)	2
(0.75,0.25)	3
(1.25,0.25)	3
(0.25,0.75)	1
(0.75,0.75)	1
(1.25,0.75)	2

**Table 2.** Training 2D RBF classifier for humidity records.

**Case** [Approximation Residual (RH%), Absolute Correlation with AP ]	**Class**
(2.5,0.25)	2
(7.5,0.25)	3
(12.5,0.25)	3
(2.5,0.75)	1
(7.5,0.75)	1
(12.5,0.75)	2

**Table 3.** Training 3D RBF classifier.

**Case** [Corr (A, B), Corr (B, C), Corr (A, C)]	**Class**
(0.25,0.25,0.25)	3
(0.25,0.25,0.75)	2
(0.25,0.75,0.25)	2
(0.25,0.75,0.75)	2
(0.75,0.25,0.25)	2
(0.75,0.25,0.75)	2
(0.75,0.75,0.25)	2
(0.75,0.75,0.75)	1
